# Ready‐to‐Use School Meals in Northern Ghana Are a Viable Alternative to Traditional School Meals

**DOI:** 10.1111/nyas.70072

**Published:** 2025-09-10

**Authors:** Issah Shani, Felix Agyemang, Donna Wegner, Angelina O. Danquah, Mark J. Manary, Kevin B. Stephenson, Firibu K. Saalia, Matilda Steiner‐Asiedu

**Affiliations:** ^1^ Department of Nutrition and Food Science University of Ghana Accra Ghana; ^2^ Project Peanut Butter Kumasi Ghana; ^3^ Department of Pediatrics Washington University St. Louis Missouri USA; ^4^ Department of Family and Consumer Sciences University of Ghana Accra Ghana; ^5^ Department of Medicine Washington University St. Louis Missouri USA

**Keywords:** Africa, Ghana, ready‐to‐use school food, school feeding, school meals

## Abstract

School feeding provides nutrition, brings order to the school day, and enhances student participation. School feeding in low‐income countries is often sporadic due to coordination challenges among multiple stakeholders. To assess the reliability of school feeding in Mion district, a food‐insecure area in northern Ghana, Project Peanut Butter (PPB) studied ready‐to‐use school meals (RUSMs) and micronutrient‐fortified home‐grown school food (HGSF). The school meals were initially provided daily in elementary schools and then extended to junior high schools. The key elements of functional programming were qualitatively compared: costs, ingredient and nutrient content, food preparation, food distribution, and consumer engagement. The cost of ingredients and nutrient content were similar between RUSM and HGSF. Safe and efficient food preparation, distribution, and storage were more readily achieved by RUSM. Consumer engagement is essential for acceptance, but can pose a challenge and disruption contingent upon the degree of ownership the community asserts over food rations. This was seen when pre‐school age children were sent to collect food rations from the elementary schools in numbers that exceeded the student enrollment. Overall, the use of a RUSM in a resource‐constrained setting allowed for greater safety and reliability of school meals at a similar cost.

## Graphical abstract

School feeding improves nutrition and attendance but faces coordination challenges in low‐income areas. In Ghana's Mion district, PPB compared RUSMs and HGSF. Both had similar costs and nutrients, but RUSMs ensured safer preparation and distribution. Community engagement was vital but sometimes disruptive, as seen when extra children collected rations. RUSMs proved more reliable in resource‐limited settings at comparable costs.

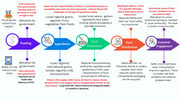

## 1 Introduction

An estimated 418 million children worldwide receive school feeding daily, though coverage varies by socioeconomic status, such that those with the greatest need are least likely to receive the benefit [1, 2, 3]. For example, only 18% of children in low‐income countries are estimated to be covered; 73 million of the most vulnerable children worldwide do not receive school meals [1, 3, 4]. School meal programs have been identified as an incentive to increase school participation and a social intervention strategy to alleviate hunger, improve the nutritional status and growth of school children, and enhance academic progression [1, 5, 6, 7, 8]. Studies have also identified a positive effect of school meals on the cognition and thus the academic performance of school children, in part depending on the ingredients used [3, 7, 9]. Evidence suggests that, in communities where school feeding programs are properly implemented, it also helps improve household food security and socio‐economic status among the beneficiaries [3, 10, 11].

Realizing the potential benefits that school meals offer, the Government of Ghana through the Ministry of Gender and Social Protection introduced the Ghana School Feeding Programme (GSFP) in 2005 [4, 12]. Its main objective was to provide meals under the home‐grown school feeding (HGSF) model to vulnerable elementary school children, while also aspiring to boost economic development in poor communities and improve household food security [4, 8, 12]. HGSF is a model wherein food is sourced locally, preferably from smallholder farmers, and individuals from the local community are contracted to cook and provide school meals [13, 14].This initiative is meant to stimulate the local community, encourage independence, and improve sustainability [13, 15]. The HGSF approach is dominant in sub‐Saharan Africa (SSA) and has been universally adopted by the Economic Community of West African States (ECOWAS) [2, 4]. While Ghana has achieved one of the highest rates of primary school enrollment in SSA, academic achievement remains low; for example, as of 2017, only 6% of second‐ or third‐graders are estimated to have achieved at least minimum proficiency in reading, and 11% in mathematics in Northern Ghana [16]. Experiences in other countries suggested that school feeding may be a powerful tool to improve nutritional and educational outcomes [2, 16, 17, 18]. Educational outcomes such as school enrollment and attendance are particularly influenced when school feeding programs are instituted in schools [19].

Despite its promise, the Ghana school meal program is challenged by low budgetary allocation, political interference, logistical constraints, and lack of adherence to the program's implementation guidelines [8, 12, 13]. Several reviews have shown that the program has largely not been able to address both nutritional and academic challenges confronting elementary schools [13, 15]. Citing delays in government payments, caterers who are hired to cook for school children do not consistently deliver meals [13, 15]. Schools earmarked as implementing schools may go full terms without the provision of a single GSFP meal [13, 14, 15]. When funding is available, low allocations limit contractors to the purchase of the cheapest locally available options, counter to the goal of school meals to improve dietary diversity among recipients. Additionally, the national buffer stock company, which has the sole responsibility of supplying food to the various schools, cannot do so because of a failure on the part of the Government to release funds promptly [8, 12, 13].

In light of the challenges of the current GSFP, Project Peanut Butter (PPB) has explored and continues to assess the use of ready‐to‐use school meals (RUSMs) in Ghanaian schools in Mion district in Northern Ghana and Atebubu in the Brong Ahafo region, respectively, across two clinical trials [9, 20]. A trial completed in 2022 was conducted at six elementary schools in which children were individually assigned to receive a peanut‐based RUSM or a locally prepared cereal porridge for one academic year [9]. A second cluster‐randomized trial, ongoing as of May 2024, includes 10 elementary and 10 junior high schools, which are assigned to receive either RUSM or cereal porridge for 24 months.

Low‐moisture, peanut‐based food aid products confer a multitude of potential advantages, including local yet centralized production, longer shelf life, and relatedly, a high degree of food safety, as well as organoleptic properties that allow for broader ingredient inclusion [21, 22]. Local, centralized production allows local ingredient sourcing, as in HGSF, yet also offers the efficiency advantages of industrialized production. In contrast to HGSF, where funds may be required at point‐of‐purchase by the caterer, leading to shortfalls when funds are not immediately available, the longer shelf life of RUSMs may allow for a more flexible payment structure for governments to achieve consistent provision of food to school children [23]. Food safety is achieved by creating a formulation with a low water content, thereby inhibiting bacterial growth as well as regulated production processes, microbiological testing, and individually sealed packaging [22, 23]. In addition, peanut‐based foods have organoleptic properties that allow for the incorporation of a variety of nutritious ingredients with strong tastes, including micronutrient powder [21, 22, 23]. Here, we report on the experience and results of the initial implementation of a RUSM program in the Mion district.

## 2 Methods

### 2.1 Theory of Ghanaian School Feeding

**FIGURE 1:**
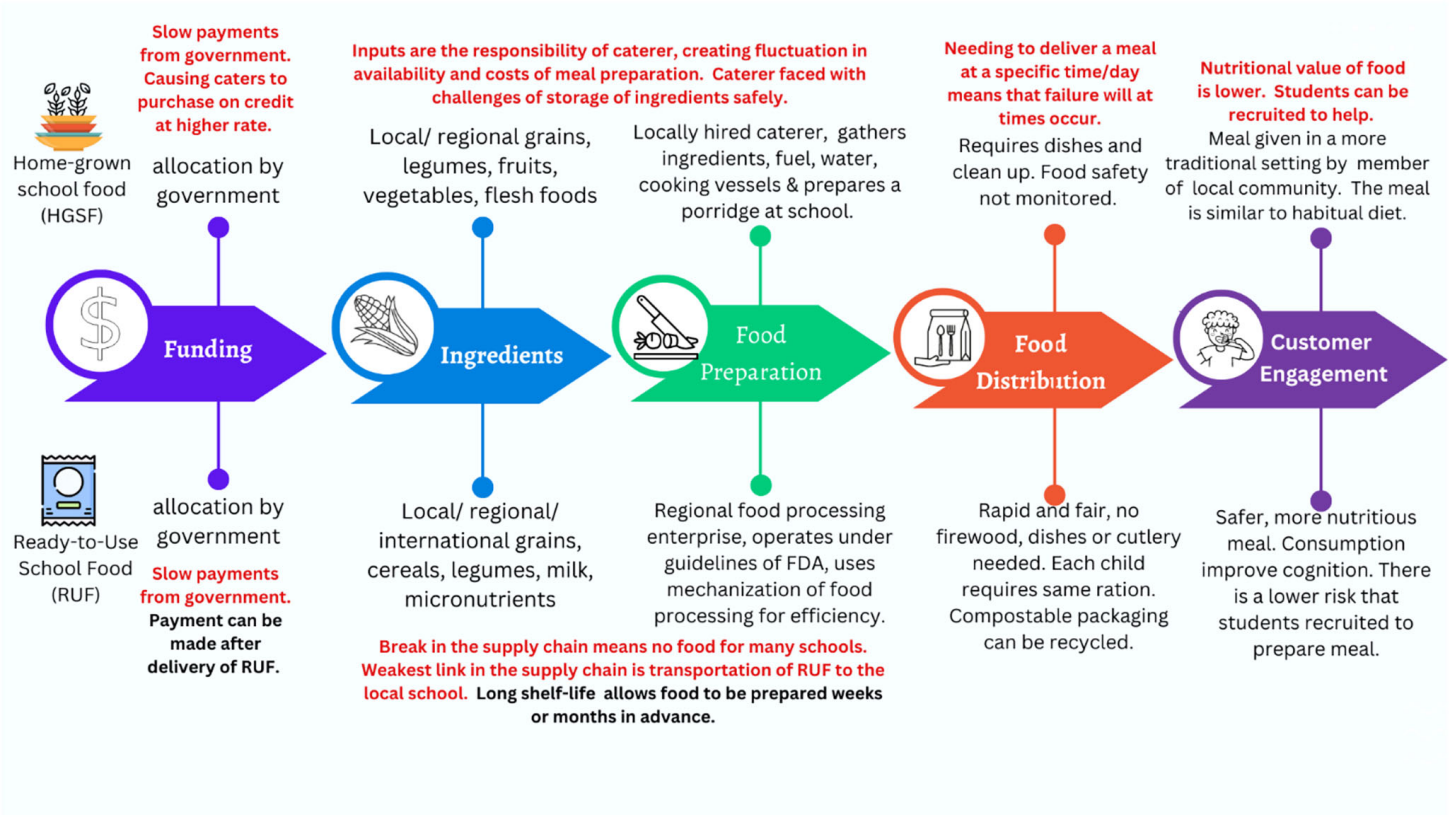
Operational elements of home‐grown school feeding and ready‐to‐use school feeding programs.

The first step in the implementation process was to develop a comprehensive operational framework of the essential elements for successful school feeding. Thirty structured group interviews were conducted in different villages. Key informants were interviewed on the implementation of the current GSFP, including school headmasters, teachers, and local village leaders, especially female community leaders. The research team then synthesized the information into a pictorial depiction shown in Figure 1, a theory of Ghanaian School Feeding. Prior work by Gelli's et al. served as a valuable reference in the creation of this depiction [24].

Figure 1 illustrates the essential operational elements of both the HGSF and RUSM programs. These include funding, ingredients, food preparation, food distribution, and customer engagement. Text in red in the figure identifies implementation barriers experienced, and text in bold identifies benefits of RUSM. The model was developed by the research team after considering the challenges and successes of school feeding in Northern Ghana over the last decade. A successful school feeding program is defined as one that is financially sustainable, delivers a meal > 80% days that school is in session, provides about 40% of the daily protein requirement of students, provides about 50% of the daily micronutrient requirements and is acceptable to the communities in the vicinity of the school.

### 2.2 Operational Setting

Mion district is a rural, Islamic area east of Tamale where most families are engaged in subsistence farming as their primary occupation [25, 26, 27]. Their diets largely consist of millet, tubers, and rice, as 92.1% engaged in cultivating and consuming these crops. Even though 94% of households in Mion rear livestock, they are more often sold rather than consumed [26]. Fruits and vegetables are infrequently consumed as only 4.7% and 16.9% are shown to consume fruits and vegetables more than 2 times a week, respectively [25, 26, 28].

Household sizes are large in the Mion district, with most of the men practicing polygamous marriage [26, 27]. Mion district is also among the most impoverished in Ghana, further reducing the availability of highly nutritious foods, such as animal‐source foods. As a result, household food and nutrition security are compromised [28]. Using the Household Food Insecurity Access Scale (HFIAS), 16% were identified as food secure or mildly insecure, and 47% were severely food insecure [29]. Previous studies have shown that inadequate food availability increases the risk of school absenteeism and dropouts and may impede the realization of the potential gains of schooling when attended [30, 31].

The district has 76 elementary schools, 29 of which are meant to be covered under the GSFP [26, 27]. Although most children attend primary school at some point, only about one‐third complete it [25, 26, 27]. Rates of formal education are low, as 25.7% of the population are currently in school, and 5.8% had at least some formal education, and thus appropriate knowledge of nutritious diets may well be limited [26, 27].

### 2.3 Selection of Study Population and Randomization

With the criterion that only one school per village be included, 20 participant schools were randomly selected, with 10 elementary schools and 10 junior high schools, by PPB staff who were unfamiliar with Mion district. Upon selection of a school, all other schools in the same community were ineligible. Schools were randomized to receive either RUSM or HGSF‐equivalent meals.

### 2.4 Outcomes

The outcomes of this study are a qualitative assessment of the five key operating elements of a school feeding program: funding, ingredients, food preparation, food distribution, and customer engagement.

### 2.5 Ethics

The study sponsors are PPB, the University of Ghana, and Washington University in St. Louis, USA. The study protocol was approved by the Ghana Health Service IRB and the Washington University Human Research Protection Office. Individual informed consent was given by each child's parent or guardian.

## 3 Results

As of May 2025, a total of 5228 children are enrolled in the ongoing study, with an average age of 13 years, of which 43% are female.

### 3.1 Funding

The purchase cost of a daily ration of RUSM without milk was $0.16, while RUSM with milk cost $0.185. The logistical cost of delivering RUSM to the schools was $31 per monthly consignment per school. The daily cost of a micronutrient fortification and an isoenergetic ration of millet was approximately $0.16.

For HGSF in Mion district, an interruption in payments means that meals will not be prepared. The proposed cost of the GSFP was USD $0.25/meal in 2016. Inflation of the local currency has reduced this government allocations to a value of $0.094/meal in 2025, far short of what is required for the caterers to provide meals meeting the national guidelines for adequate nutrition. For HGSF, all funds must first be transferred into the account of the local caterers, and then those funds are used to procure foodstuffs and ingredients for cooking. This process often leads to inconsistent provision of food for school children.

### 3.2 Ingredients of RUSM and HGSF

The RUSM was composed of peanut, cowpea, palm oil, sugar, milk, and maize. These macronutrient ingredients were sourced from local Ghanaian markets. The macronutrient contents of the three foods are shown in Table 1. Inclusion of milk powder increases protein intake 1.5‐fold. Both RUSM and millets were supplemented with micronutrients as shown in Table 2. Each school meal required micronutrient fortification because neither of them provided sufficient micronutrients based on ingredients alone. While it has been suggested that HGSF may allow for greater dietary diversity [10], the choice of food is limited by the cost, which introduces monotony.

**TABLE 1:** The nutritional content of the three food formulations used for the school meal.

Nutrient	Ready‐to‐use school meal: milk	Ready‐to‐use school meal: vegetable	Millet porridge
Energy (kcal)	402	416	412
Protein (g)	17.6	13.5	11.9
Fat (g)	24.7	28.9	5.4
Essential Amino Acids (g)	7.13	4.78	5.16

**TABLE 2:** Micronutrient composition of the food formulations.

Micronutrient	Ready‐to‐use school meal: vegetable	Ready‐to‐use school meal: milk	Millet porridge	Micronutrient fortification	Recommended daily allowance for 4–8‐year‐old children
Vitamin A (µg)	3	10	0	189	400
Vitamin C (mg)	0.2	14	0	17.3	25
Vitamin D (µg)	0	0	0	3.2	15
Vitamin E (mg)	2.0	1.6	2.7	4.1	7
Vitamin K (µg)	0.2	0.40	1	5.2	55
Thiamin (mg)	0.2	0.19	0.41	0.28	0.6
Riboflavin (mg)	0.32	0.51	0.07	0.50	0.6
Niacin (mg)	7.0	5.3	6.0	3.0	8
Vitamin B6 (mg)	0.2	0.23	0.37	0.43	0.6
Folate (µg)	143	148	42	58.7	200
Vitamin B12 (µg)	0	1.0	0	0.59	1.2
Pantothenic acid (mg)	0.7	0.52	1.3	0.32	2
Biotin (µg)	32	25	0	12.6	12
Calcium (mg)	31	309	14	32.4	1000
Copper (µg)	134	163	535	216	440
Iodine (µg)	0	0	0	20	90
Iron (mg)	2.5	1.6	3.9	1.8	10
Selenium (µg)	9.3	6.7	33	2.7	30
Zinc (mg)	2.1	1.5	2.6	2.0	5.0

### 3.3 Food Preparation

The RUSM was prepared by PPB in its factory in Kumasi, following the requirements of the Food and Drug Administration of Ghana and the CODEX Alimentarius to produce low‐moisture foods. The peanuts were sourced from the local market, tested for aflatoxins, and if aflatoxin was < 20 ppb, they were roasted. Cowpea was also roasted and milled. All other ingredients were added to the peanut paste to formulate the RUSM food. When distributed at school, no preparation of the RUSM was required. These foods were prepared with a long shelf life to mitigate any form of spoilage after delivery to schools. Therefore, stock‐outs of RUSM did not occur during the implementation period.

For the HGSF meals, raw materials (millet and sugar) were purchased locally from the district and supplied to caterers in the schools. Millet powder was used to prepare porridge and served to school children on each school day. The quantity of millet provided to each school was based on the number of children enrolled. Usually, two 50‐kg bags of locally sourced millet were provided, along with 50 kg of white sugar to last for 10‐school days.

### 3.4 Food Distribution

All the students at the included schools were offered a daily meal, either HGSF or RUSM. Meals were consumed avidly and completely in almost every instance. RUSM was provided to schools fortnightly. The quantity was adjusted according to the children enrolled at each school. Foodstuffs for the HGSF were quantified and delivered to each school in quantities to last for 2 weeks. Caterers were responsible for keeping these foodstuffs in good condition and ensuring their safe preparation. Logistical challenges encountered at HGSF schools included ingredient availability, preparation, surveillance, and distribution of the school meals. Caterers had to prepare meals on site every school day. Firewood, clean water, ingredients and dishes are required to serve the food. Supervision of caterer meal provision was time‐consuming, as were questions and comments from caterers, frequently regarding ingredient amounts. In contrast, the requirements for RUSMs required only school headmaster and teacher engagement to properly store and dispense the food.

RUSM retained a shelf life of 12 months at ambient temperatures in Mion, averaging 29°C. The RUSM packaging maintained its microbial safety and protected it from insects. RUSM was stored in cardboard boxes placed in small wire mesh cupboards at the schools to limit exposure to rodents and larger animals. HGSF meals use fresh ingredients purchased in local markets, so food safety for HGSF meals is established at the time of cooking. Uncooked cereals were stored in large, sturdy plastic buckets and cans, which provided sufficient protection from birds and rodents.

### 3.5 Community/Consumer Engagement

All communities in the Mion district accepted the distribution of RUSM and HGSF meals at their schools. The acceptability of the school food was evident, as parents, caregivers, and teachers called for the extension of the intervention period when its time was due.

During the 2021–2022 clinical trial, school attendance was recorded manually by teachers. In the current trial, attendance is tracked biometrically daily with a fingerprint. The amount of food delivered by PPB is determined using the biometric attendance data. Soon after implementation, participating schools complained that inadequate amounts of food were being delivered by PPB.

A monitor was sent to each school to count the number of children receiving food for a week. While approximately 5000 elementary children attended school, 12,310 collected a school meal. For junior high students, 1032 children attended school while 1080 children collected a meal. This discrepancy in elementary school was attributed to younger children from the community who were not enrolled in school seeking a meal. Discussions with community leaders revealed that many community members felt a sense of ownership of the activities at elementary schools, including meal distribution, and that school activities should and could be modified to make them more acceptable to the community at large. It was not generally accepted that the meals should be reserved for children attending the school.

There was high acceptance of the RUSM feeding model in all communities where the implementation took place. Students' acceptability was particularly high as they warmly embraced all the food formulations that were provided.

## 4 Discussion

The provision of a RUSM in elementary schools in northern Ghana was an acceptable and effective means to provide a meal. This is evident as many teachers in other schools plead with the University of Ghana and PPB to extend the RUSM model to their respective schools. All the headmasters reported that attendance had increased and that some students who had not been in school started attending.

A limitation of this study is that it provides little quantitative data. The information is reported as a qualitative description. This study design was chosen to avoid perturbing the behavior surrounding food management and distribution. The Hawthorne effect has been shown to bias food consumption studies in multiple contexts previously. The lack of individual observations only allows for conclusions that are seen in a large majority of participants and schools. This differs from the individually randomized, controlled clinical trial our group conducted in 2021–2022 [9]. The 2022 study had a much smaller sample size in terms of participants and the number of schools. The qualitative description allowed for the detection of program variations that will arise during operation.

The costs of the ingredients in HGSF and RUSM were similar, as was nutrient intake. Both foods were largely made with local ingredients, which were sourced from the Mion district. Thus, with HGSF and RUSM, school feeding supported the local agricultural economy. This conforms with the principles of HGSF models [15, 32].

The program alleviated the plight of parents and guardians in providing food or money for the upkeep of their children in school. It also offered the opportunity for teachers to have the full complement of their various classes for teaching and learning activities to take place. They indicated that, previously, the lack of feeding programs in the various schools has prompted children to drop out of school, as they always complain of the inability to get food to eat during school hours. This is evident as both teachers and parents advocated for the extension of the program when its time was due.

The RUSM was more reliable than HGSF due to its longer shelf life, disengagement of food preparation from meal consumption, and providing the opportunity of adding a high‐quality ingredient such as milk in a cost‐effective manner.

The community viewed the provision of a meal at elementary schools as a service that should not be limited to just students but open to all children. This is consistent with other findings reported elsewhere [33, 34]. The high level of food insecurity and traditional subsistence agrarian structure of the community likely fosters this belief and structures the practice of school feeding. This may also reduce the health and educational benefits for those enrolled in school. Novel efforts to target school meals are needed to maximize the educational benefit while not alienating the supportive community. The HGSF model prepares food at a single location, usually on the grounds of the school. The addition of a community‐based preschool feeding program in combination with a RUSM for students may enhance community acceptance.

RUSM presents a viable alternative to HGSF for elementary students in the Mion district. Because RUSM included multiple nutrient‐dense ingredients, it improved dietary diversity when compared to HGSF. RUSM was more reliable food and not subject to interruptions due to the availability of cooking fuel and utensils that beset HGSF. RUSM availability was more consistent by extension of financial credit by food producers to the primary payer, the Government, while HGSF is directly reliant on multiple, frequent payments to caterers. A comparison of RUSM and HGSF regarding the benefits to learning and school achievement is warranted to better assess which method best affects the desired outcome.

## 5 Conclusion

This study demonstrates that RUSM presents a viable alternative to traditional HGSF in resource‐constrained settings. The RUSM model addressed key operational challenges, including food safety, consistent availability, and cost‐effectiveness, while maintaining community acceptability. However, the finding that non‐enrolled children collected meals highlights the need for complementary community feeding programs. Governments across sub‐Saharan Africa should consider piloting RUSM programs, particularly in areas where HGSF faces persistent implementation challenges. Future research should evaluate the comparative impact of RUSM versus HGSF on educational and health outcomes through longer‐term studies.

## Author Contributions

Issah Shani, Donna Wegner, Mark J. Manary, Kevin B. Stephenson, Firibu K. Saalia, and Matilda Steiner‐Asiedu contributed to the conception and design of the project. Issah Shani, Felix Agyemang, Donna Wegner, Mark J. Manary, Kevin B. Stephenson, Firibu K. Saalia, and Matilda Steiner‐Asiedu implemented the work and collected the data. Issah Shani, Mark J Manary, Angelina O. Danquah, Kevin B. Stephenson, and Matilda Steiner‐Asiedu analyzed the information. Issah Shani wrote the first draft of the manuscript. All the authors had revised the manuscript and approved its final content.

## Conflicts of Interest

The authors declare no conflicts of interest.

## 1 Peer Review

The peer review history for this article is available at https://publons.com/publon/10.1111/nyas.70072.

## Data Availability

The data that support the findings of this study are available on request from the corresponding author. The data are not publicly available due to privacy or ethical restrictions.
